# Correlation between serum uric acid and body fat distribution in patients with MAFLD

**DOI:** 10.1186/s12902-023-01447-7

**Published:** 2023-09-25

**Authors:** Min Tao, Jing Liu, Xingyu Chen, Qing Wang, Miao He, Wenwen Chen, Cong Wang, Lili Zhang

**Affiliations:** Department of Endocrinology, the Second Affiliated Hospital, Chongqing Medical University, Chongqing, 400010 China

**Keywords:** Metabolic dysfunction associated with fatty liver disease, Hyperuricemia, Visceral fat, Obesity

## Abstract

**Background:**

Metabolic dysfunction associated with fatty liver disease (MAFLD) is often correlated with obesity and hyperuricemia. The present study aimed to determine the association between serum uric acid (SUA) and central fat distribution in patients with MAFLD.

**Methods:**

A total of 485 patients were classified into the following groups: (1) controls without MAFLD and hyperuricemia (HUA), (2) MAFLD with normal SUA, and (3) MAFLD with HUA. DUALSCAN HDS-2000 was used to measure visceral fat (VAT) and subcutaneous fat (SAT). Dual-energy X-ray absorptiometry (DEXA) was used to measure body fat distribution.

**Results:**

MAFLD patients with HUA had remarkably higher BMI, fasting insulin, OGIRT AUC, ALT, AST, TG, VAT, SAT, Adipo-IR, trunk fat mass, android fat, and total body fat than MAFLD patients with normal SUA (all *p* < 0.05). The increase in VAT, SAT, CAP, Adipo-IR, upper limbs fat mass, trunk fat mass, and android fat, as well as the percentage of MAFLD, were significantly correlated with the increase in SUA. The percentage of MAFLD patients with HUA increased significantly with increasing VAT or SAT, as determined by the Cochran–Armitage trend test (all *p* < 0.05). Furthermore, VAT (OR = 1.01 CI: 1.00, 1.03; *p* < 0.05) and adipo-IR (OR = 1.09 CI: 1.00, 1.19; *p* < 0.05) were associated with circling SUA in MAFLD after adjusting for sex, age, TG, TC, HOMA-IR, and BMI.

**Conclusion:**

Abdominal fat promotes the co-existence of HUA and MAFLD, while weight loss, especially, decreasing VAT, is of great importance to decrease SUA levels and manage MAFLD.

## Introduction

MAFLD redefines fatty liver disease by focusing on metabolic abnormalities (irrespective of the underlying cause of chronic liver disease) and their pivotal role in the clinical outcomes of individuals with hepatic steatosis [1, 2]. It often parallels the prevalence of obesity and affects approximately 70–90% of overweight or obese patients [3]. Patients with MAFLD often experience other metabolic disorder complications, including type 2 diabetes mellitus (T2DM), hyperlipidemia, metabolic syndrome, and hyperuricemia [4, 5], as well as, an increased risk of cardiovascular disease (CVD) and related mortality. Although not every patient with hyperuricemia suffers from gout, hyperuricemia is a major etiologic factor for gout [6]. Furthermore, hyperuricemia has been strongly correlated with several metabolic diseases, like T2DM [7–9], metabolic syndrome [10–12] hypertension [13, 14], and CVD [11, 15–17]. Serum uric acid (SUA) has been frequently found to be significantly higher in morbidly obese patients compared to controls [18]. Elevated SUA is consistent with increasing metabolic characteristics and positively correlated with abdominal fat [18, 19]. Visceral fat has been reported as the most influential factor in hyperuricemia [19]. Moreover, the decrease in SUA level is positively associated with reduced visceral fat area in gout patients [20]. Various epidemiological and clinical studies have reported an association between hyperuricemia and Nonalcoholic fatty liver disease (NAFLD) [21–23]. Hyperuricemia has been shown to independently be related to both hepatic and visceral fat tissue quantified by computer tomography [19, 24]. A recent meta-analysis suggested that hyperuricemia is associated with an exacerbated risk of NAFLD [25]. However, to the best of our knowledge, no studies have evaluated the correlation between body fat distribution and SUA in patients with MFLAD. Therefore, this study aimed to determine the association between body fat distribution and SUA in MAFLD patients.

## Patients and methods

### Participants

**Fig. 1:**
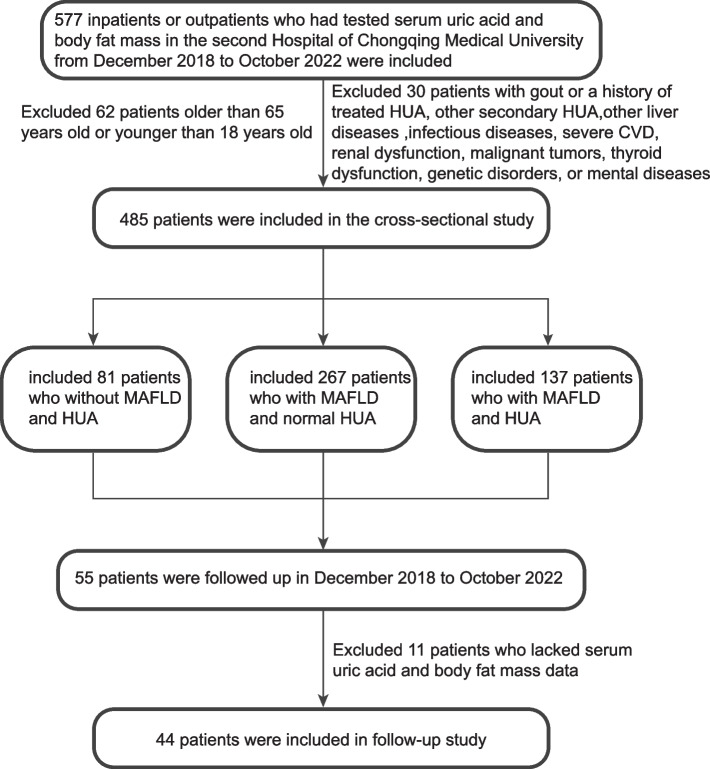
Flow chart of the study population

Four hundred and eighty-five individuals (Including 260 males and 225 females) were enrolled in this study from inpatient or outpatient at the Second Affiliated Hospital of Chongqing Medical University from December 2018 to October 2022. The participants were divided into the following three groups (Fig. 1): (1) normouricemic [controls without MAFLD and hyperuricemia (HUA)], *n* = 81; (2) MAFLD with normal SUA, *n* = 267; and (3) MAFLD with HUA, *n* = 137. The present study was approved by the Ethics Committee of our hospital (Chongqing, China) and conducted in accordance with the Declaration of Helsinki. Written informed consent was obtained from all participants in the study.

Hyperuricemia was defined as SUA over 420 umol/L [26, 27]. MAFLD was diagnosed according to the criteria reported by An international experts group [28].

All of the participants enrolled were aged 18–65 years. The exclusion criteria for the present study were as follows: i) patients with gout or a history of treated HUA, or other secondary HUA, ii) patients with other liver diseases and infectious diseases, severe CVD, renal dysfunction, malignant tumors, thyroid dysfunction, genetic disorders, or mental diseases, or under any medication that affects the present study.

### Clinical and laboratory measurements

After 10–12 h of overnight fasting, peripheral blood samples were collected between 7 and 9 am. Anthropometric parameters, such as weight and height, were measured according to standardized protocols as done in our previous study [29]. Systemic systolic blood pressure (SBP) and diastolic blood pressure (DBP) were obtained by a designated nurse using a mercury sphygmomanometer, and the average of three consecutive measurements was considered. Body mass index (BMI) was calculated as weight (Kg) divided by the square of height (m^2^). The calculation of waist-to-hip ratio (WHR) involved dividing the waist circumference (in centimeters) by the hip circumference (in centimeters) [30]. Moreover, the WHR was categorized into two groups: android, characterized by a WHR of ≥ 0.85, and gynoid, indicated by a WHR of < 0.85. Liver steatosis by controlled attenuation parameter [CAP (dB/m)] was measured by liver ultrasonic attenuation at 3.5 MHz [31]. The levels of triglycerides (TG), total cholesterol (TC), high-density lipoprotein cholesterol (HDL-C), low-density lipoprotein cholesterol (LDL-C), free fatty acids (FFAs), aspartate aminotransferase (AST), alanine aminotransferase (ALT), and uric acid (UA) were determined by standard enzymatic assays as in previous study [32]. Glycosylated hemoglobin (HbA1c) was measured by ion-exchange high-performance chromatography and plasma glucose was measured-using the glucose oxidase method. Plasma insulin was measured by chemiluminescence. The homeostasis model assessment of insulin resistance (HOMA-IR) was calculated as fasting plasma glucose (FPG, mmol/L) × fasting insulin (FINS, mU/L)/22.5 [33]. Adipose tissue insulin resistance (Adipo-IR) was calculated as free fatty acid (FFA, mmol/L) × fasting insulin (FINS, mU/L) [34]. The NAFLD score (NFS) was calculated as—1.675 + 0.037 × age (years) + 0.094 × BMI (kg/m^2^) + 1.13 × impaired fasting glucose/diabetes (yes = 1, no = 0) + 0.99 × AST/ALT ratio-0.013 × platelet count (10^9^/L)—0.66 × albumin (g/dL) [35]. Fibrosis-4 (FIB-4) was calculated as age (years) × AST (U/L)/[platelet count (10^9^/L) × ALT^1/2^ (U/L)] [36].

### Oral glucose insulin release test (OGIRT)

The 75 g glucose-OGIRT test was conducted after 10-12 h of fasting in all participants. At 8 a.m. on the test day, all participants were given 75 g of oral glucose, and blood samples were collected at designated time points (0, 30, 60, and 120 min) to measure insulin.

### Abdominal fat measurement

Visceral and subcutaneous fat were evaluated by Dual bioelectrical impedance (DUALSCAN HDS-2000) with the patient in a supine position on an empty stomach, in a quiet state, by a dedicated operator. A dual BIA instrument was used to calculate the cross-sectional area of visceral and subcutaneous fat at the level of the umbilicus based on the measurement of electrical potentials in two different body spaces, as previously described [37].

### Body fat distribution measurements

Body fat distribution in patients with MAFLD was assessed by dual-energy X-ray absorptiometry (DEXA, APEX 4.5.0.2, Hologic, USA). The fat mass was measured in the whole body including the head, trunk, android, upper limbs, thighs, and gynoid regions. The percentage of android fat and percentage of gynoid fat ratio (A/G ratio) was calculated.

### Statistics

Statistical analyses were performed using SPSS statistical software (Version 26.0, SPSS Inc., Chicago, Illinois, USA). Shapiro–Wilk test was used to detect normally distributed data. Normally distributed data are represented as the mean ± standard deviation (SD) while data showing skewed distribution are represented as the median (interquartile range). Variables with a normal distribution were analyzed by ANOVA was used to compare among groups and the Bonferroni method was used to test to differences between groups; skewed distribution variables were analyzed by the Kruskal–Wallis 1-way test and the Mann–Whitney U test was used to compare variables between the certain two groups. In the pooled data, SUA was divided into quartiles and the Cochran–Armitage trend test was used to estimate the significant trends across increasing quartiles. The chi-squared test was used to analyze categorical data between different groups. Pearson correlation was used to determine the correlation of data. Unilinear and logistic regression analyses were performed to explore the correlation between SUA and fat distribution in MAFLD. Besides, we divided the visceral fat and subcutaneous fat into quartiles and used the Cochran–Armitage trend test to assess the significant trends through increasing quartiles. The two-tailed *P* values < 0.05 were considered statistically significant. For all statistical tests, *P* values < 0.05 were considered statistically significant.

## Results

### Basic characteristics of the patients in different groups

Table 1 shows the basic characteristics the anthropometric, metabolic characteristics, and body fat distribution of the patients in the three groups. Compared to the control group, participants in the MAFLD with normal SUA and MAFLD with HUA groups had higher Weight, BMI, WHR, SBP, DBP, fasting insulin, ALT, AST, TG, VAT, SAT, CAP, FFA, Adipo-IR, trunk fat mass, android fat, total body fat, and lower HDL-C (all *P* < 0.05). As expected, the MAFLD with the HUA group had remarkably higher Weight, BMI, fasting insulin, OGIRT AUC, ALT, AST, TG, VAT, SAT, Adipo-IR, trunk fat mass, android fat mass, and total body fat than the MAFLD with normal SUA group (all *P* < 0.05). There were no statistically significant differences in TC, LDL-C, NFS, FIB-4, or numbers of metabolic syndromes between the MAFLD with HUA or with normal SUA groups. Furthermore, there were no statistically significant differences in WHR, SBP, DBP, HDL-C, CAP, and FFA (*P* > 0.05) between the two groups.

**Table 1:** Anthropometric measurements, metabolism and body fat distribution of patients in different groups

Items	normouricemic	MAFLD with normal SUA	MAFLD with high SUA	*P* values
	***N* = 81**	***N* = 267**	***N* = 137**	
**Age (years)**	40.58 ± 11.55	42.50 ± 11.03	34.33 ± 10.04	** < 0.000**^**b,c**^
**Weight (kg)**	63.01 ± 11.72	74.37 ± 11.67	85.45 ± 15.71	** < 0.000**^**a,b,c**^
**BMI (kg/m**^**2**^**)**	24.14 ± 3.15	27.67 ± 3.85	30.49 ± 4.76	** < 0.000**^**a,b,c**^
**WHR**	0.87 (0.84,0.92)	0.93 (0.88,0.97)	0.95 (0.89,0.99)	** < 0.000**^**a,b**^
**SBP (mmHg)**	119 (107.75,132.00)	130.00 (122.00,140.00)	132.00 (121.00,142.00)	** < 0.000**^**a,b**^
**DBP (mmHg)**	75.67 ± 12.27	80.89 ± 10.43	82.57 ± 10.55	** < 0.000**^**a,b**^
**Ogirt 0 min (uU/mL)**	8.17 (4.39,11.93)	14.62 (9.29,22.33)	22.23 (15.35,35.53)	** < 0.000**^**a,b,c**^
**Ogirt AUC**	3988.80 (2039.25, 7219.35)	7390.20 (2890.05,13,046.40)	11,586.45 (5683.50,20,019.50)	** < 0.000**^**b,c**^
**ALT (U/L)**	18.00 (13.00,24.00)	26.00 (19.00,40.00)	43.00 (25.00,82.50)	** < 0.000**^**a,b,c**^
**AST (U/L)**	18.00 (14.00,22.00)	20.00 (16.00,29.00)	29.00 (19.00,52.00)	** < 0.000**^**a,b,c**^
**TC (mmol/L)**	4.92 (4.38,5.69)	5.04 (4.38,5.87)	5.29 (4.65,6.25)	0.114
**TG (mmol/L)**	1.40 (0.93,2.25)	1.93 (1.43,3.41)	2.49 (1.72,4.26)	** < 0.000**^**a,b,c**^
**LDL-c (mmol/L)**	2.63 (2.22,3.21)	2.70 (2.15,3.21)	2.74 (2.20,3.37)	0.804
**HDL-c (mmol/L)**	1.19 ± 0.28	1.08 ± 0.26	1.05 ± 0.24	**0.001**^**a,b**^
**SUA (μmol/L)**	281.15 ± 72.52	322.84 ± 59.97	502.66 ± 75.94	** < 0.000**^**b,c**^
**VAT (cm**^**2**^**)**	61.00 (40.50,79.00)	95.00 (76.00,114.00)	112.00 (92.00,137.00)	** < 0.000**^**a,b,c**^
**SAT (cm**^**2**^**)**	155.51 ± 73.25	213.52 ± 76.86	263.98 ± 85.00	** < 0.000**^**a,b,c**^
**NFS**	-1.77 ± 2.27	-1.86 ± 1.86	-2.25 ± 2.23	0.163
**FIB-4**	0.79 (0.52,1.19)	0.79 (0.56,1.19)	0.70 (0.46,1.16)	0.194
**HOMA-IR**	1.44 (1.18796,2.75748)	2.80 (1.36,6.04)	2.34 (1.21,5.93)	**0.011**^**a**^
**CAP**	202.86 ± 23.77	301.38 ± 37.24	316.70 ± 39.18	** < 0.000**^**a,b**^
**FFA (mmol/L)**	0.45 ± 0.28	0.56 ± 0.23	0.64 ± 0.22	**0.001**^**a,b**^
**Adipo-IR**	2.42 (0.10,4.3407)	6.53 (3.14,11.69)	12.72 (5.98,21.93)	** < 0.000**^**a,b,c**^
**metabolic syndrome (%, n)**	45.70 (37.00)	64.00 (171.00)	73.00 (100.00)	0.17
**Total body fat (%)**	38.05 (35.78,40.40)	42.10 (38.35,45.65)	40.90 (35.40,45.00)	**0.035**^**a**^
**Upper limbs fat mass (g)**	3068.86 ± 812.91	3391.38 ± 775.40	3727.95 ± 682.82	**0.018**^**b**^
**Thighs fat mass (g)**	7383.50 (6082.00,8327.25)	8607.50 (6914.75,10,142.50)	9422.00 (7489.00,12,372.00)	**0.017**^**b**^
**trunk fat mass (g)**	12,534.00 (11,860.50,14,263.25)	17,726.00 (15,563.00,20,313.80)	20,288.00 (17,032.00,24,411.00)	** < 0.000**^**a,b,c**^
**Android (g)**	1900.50 (1661.50,2172.75)	2870.50 (2535.75,3382.75)	3434.00 (2710.00,4151.00)	** < 0.000**^**a,b,c**^
**Gynoid (g)**	4052.50 (3549,00,4263.00)	4704.00 (4112.25,5501.00)	5165.00 (4013.00,6522.00)	**0.005**^**b**^
**Total Body fat (g)**	25,165.93 ± 6476.60	31,210.53 ± 5594.23	36,288.14 ± 8080.05	** < 0.000**^**a,b,c**^

Normally distributed data are represented as the mean ± standard deviation (SD), while data showing skewed distribution are represented as the median (interquartile range)

*Abbreviations*: *BMI* body mass index, *WHR* waist hip ratio, *SBP* systolic blood pressure, *DBP* diastolic blood pressure, *Ogirt* oral glucose insulin releasing test, O*girt AUC* area under Ogirt curve, *ALT* alanine aminotransferase, *AST* aspartate aminotransferase, *TC* total cholesterol, *TG* triglyceride, *LDL-c* low-density lipoprotein cholesterol, *HDL-c* high-density lipoprotein cholesterol, *SUA* serum uric acid, *VAT* visceral adipose tissue, *SAT* subcutaneous adipose tissue, *NFS* Non-alcoholic fatty liver disease fibrosis score, *FIB-4* Fibrosis-4 score, *HOMA-IR* homeostasis model assessment of insulin resistance, *CAP* Controlled attenuation parameters, *FFA* free fatty acid, *Adipo-IR* adipose tissue insulin resistance

^a^indicates that there is a statistically significant difference between normouricemic and MAFLD with normal SUA

^b^indicates that there is a statistically significant difference between normouricemic and MAFLD with high SUA

^c^indicates that there is a statistically significant difference between MAFLD with normal SUA and MAFLD with high SUA

### Comparison of fat distribution, prevalence of MAFLD, and metabolic syndrome (MS) across the quartile of SUA

We established that compared with MAFLD patients with normal SUA, those with HUA have much higher abdominal fat and are more insulin resistant, especially with a higher adipo-IR. We then mainly focused on determining how the fat distribution and prevalence of MAFLD changed with different levels of uric acid (Table 2). As expected, the BMI, WHR, fasting insulin, OGIRT AUC, VAT, SAT, CAP, Adipo-IR, upper limbs fat mass, trunk fat mass, and android fat mass was significantly increased with increasing SUA and the prevalence of MAFLD was also increased with increasing quartiles of SUA. Furthermore, the percentage of MS also significantly increased with increasing quartiles of SUA. However, no significant difference in percent total body fat, gynoid fat mass, and thighs fat mass was observed across various SUA quartiles.

**Table 2:** Anthropometric measurements, metabolism and body fat distribution of patients across quartiles of serum uric acid

Items	Quartile of SUA				*P* values
	**Q1**	**Q2**	**Q3**	**Q4**	
**n**	120.00	118.00	126.00	121.00	
**BMI (km/m**^**2**^**)**	25.04 ± 3.78	27.07 ± 3.99	28.63 ± 4.01	30.58 ± 5.06	< 0.000
**WHR**	0.90 (0.86,0.96)	0.92 (0.86,0.96)	0.93 (0.87,0.98)	0.95 (0.90,0.99)	< 0.000
**Ogirt 0 min (uU/L)**	11.32 (6.07,17.10)	12.85 (7.29,19.73)	16.19 (10.17,22.22)	22.96 (15.77,35.74)	< 0.000
**Ogirt AUC**	4965.00 (1967.85,8721.04)	6105.75 (2659.8,012100.50)	9752.10 (4284.68,13,872.38)	11,823.90 (5992.50,21,401.33)	< 0.000
**SUA (umol/L)**	240.43 ± 41.14	324.66 ± 15.62	389.77 ± 22.87	517.27 ± 78.95	< 0.000
**VAT (cm**^**2**^**)**	74.00 (53.50,99.50)	88.00 (62.50,105.00)	99.00 (69.50,123.00)	112.00 (89.50,137.50)	< 0.000
**SAT (cm**^**2**^**)**	166.21 ± 72.04	202.25 ± 74.08	239.41 ± 90.90	261.03 ± 89.52	< 0.000
**CAP**	262.83 ± 59.27	273.84 ± 51.33	291.21 ± 51.63	314.65 ± 43.65	< 0.000
**FFA (mmol/L)**	0.50 ± 0.27	0.52 ± 0.22	0.571 ± 0.22	0.64 ± 0.23	0.003
**Adipo-IR**	4.40 (1.80,7.34)	4.52 (2.59,10.21)	6.98 (4.90,10.22)	12.01 (6.89,21.93)	< 0.000
**Total body fat (%)**	41.80 (37.20,44.70)	41.10 (37.60,44.60)	41.70 (38.35,45.60)	40.40 (35.05,43.60)	0.539
**Upper limbs fat mass (g)**	3204.31 ± 839.87	3262.85 ± 707.87	3456.07 ± 768.26	3734.08 ± 727.41	0.027
**Thighs fat mass (g)**	8633.00 (7509.75,10,137.25)	8219 (6787.50,9699.50)	8601.00 (6304.00,11,051.00)	9194.50 (7010.75,12,246.50)	0.710
**trunk fat mass (g)**	16,450.00 (14,886.00,20,252.50)	16,351.00 (14,178.00,19,070.50)	18,103.00 (15,421.50,21,558.50)	20,700.00 (17,234.25,23,629.00)	< 0.000
**Android fat mass (g)**	2642.00 (2246.25,3361.25)	2547.00 (2128.00,3014.00)	2933.00 (2548.50,3564.50)	3535.50 (2735.50,4103.00)	< 0.000
**Gynoid fat mass (g)**	4650.00 (4158.25,5491.25)	4362.00 (3859.50,5172.50)	5036.00 (3774.00,5993.00)	4918.50 (3982.25,6268.50)	0.296
**Total Body fat (g)**	30,094.75 ± 6440.41	29,273.58 ± 5409.94	32,194.39 ± 7015.48	35,921.40 ± 8468.93	0.001
**Fatty Liver disease (%, n)**	(62.50) 75.00	(82.20) 97.00	(87.30) 110.00	(92.60) 112.00	< 0.000
**Metabolic syndrome (%, n)**	(40.80) 49.00	(60.20) 71.00	(70.60) 89.00	(81.00) 98.00	< 0.000

Normally distributed data are represented as the mean ± standard deviation (SD), while data showing skewed distribution are represented as the median (interquartile range)

*Abbreviations*: *BMI* body mass index, *WHR* waist hip ratio, *Ogirt* oral glucose insulin releasing test, *Ogirt AUC* area under Ogirt curve, *SUA* serum uric acid, *VAT* visceral adipose tissue, *SAT* subcutaneous adipose tissue, *CAP* Controlled attenuation parameters of liver, *FFA* free fatty acid, *Adipo-IR* adipose tissue insulin resistance

### Correlations between fat distribution and SUA

**Fig. 2:**
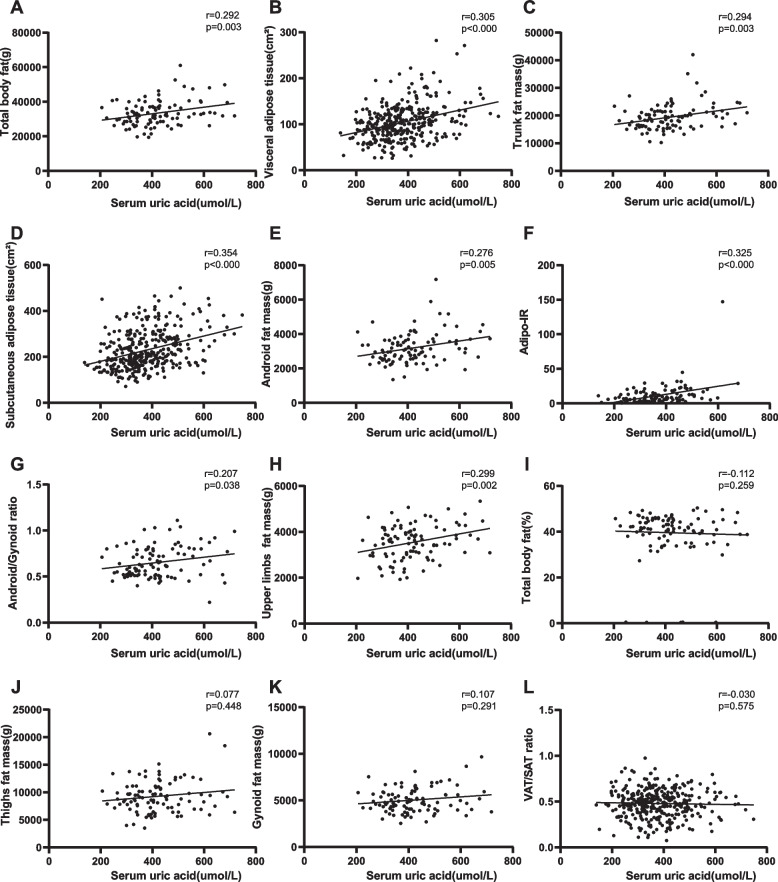
Correlation between SUA level and fat in different regions. **A** Correlation between serum uric acid and total body fat. **B** Correlation between serum uric acid and visceral adipose tissue. **C** Correlation between serum uric acid and trunk fat mass. **D** Correlation between serum uric acid and subcutaneous adipose tissue. **E** Correlation between serum uric acid and android fat mass**. F** Correlation between serum uric acid and Adipo-IR. **G** Correlation between serum uric acid and android/gynoid ratio. **H** Correlation between serum uric acid and upper limbs fat mass. **I** Correlation between serum uric acid and total body fat (%). **J** Correlation between serum uric acid and Thighs fat mass. **K** Correlation between serum uric acid and gynoid fat mass. **L** Correlation between serum uric acid and VAT/SAT ratio

The altered fat distribution and percentage of MAFLD across different circulating levels of SUA prompted us to explore the correlation between different fat deposits and SUA. As shown in Fig. 2, We found that SUA levels were significantly and positively associated with total fat mass (*r* = 0.292, *p* < 0.01), VAT (*r* = 0.305, *p* < 0.01), trunk fat mass (*r* = 0.294, *p* < 0.01), SAT (*r* = 0.354, *p* < 0.01) android fat (*r* = 0.276, *p* < 0.01), Adipo-IR (*r* = 0.325, *p* < 0.01), ratio of android and gynoid fat (*r* = 0.207), *p* < 0.05) and upper limbs fat mass (*r* = 0.299, *p* < 0.01). However, we didn’t find any significant correlation between SUA and percent of total body fat, thighs fat mass, gynoid fat mass, and the ratio of VAT and SAT. Next, we performed logistic regression analysis to explore variables that had independent associations with circulating SUA in MAFLD (Table 3). After adjusting for sex, age, blood pressure, and BMI, VAT (OR = 1.016 CI: 1.004, 1.027 *p* = 0.006) and adipo-IR (OR = 1.089 CI: 1.017, 1.166 *p* = 0.014) were still significant risk factors for SUA in MAFLD. After adjusting for sex, age, HbA1c, and BMI, VAT (OR = 1.018 CI: 1.004, 1.032, *p* = 0.009) and adipo-IR (OR = 1.079 CI: 1.006, 1.157, *p* = 0.033) were still significant risk factors for SUA in MAFLD. After adjusting for sex, age, HOMA-IR, and BMI, VAT (OR = 1.015 CI: 1.002, 1.027, *p* = 0.023) and adipo-IR (OR = 1.100 CI: 1.010, 1.198, *p* = 0.028) were still significant risk factors for SUA in MAFLD. Furthermore, after adjusting for sex, age, TG, TC, HOMA-IR, and BMI, VAT (OR = 1.014 CI: 1.001, 1.028, *p* = 0.032) and adipo-IR (OR = 1.094 CI: 1.002, 1.194, *p* = 0.046) were still independent risk factors for SUA in MAFLD.

**Table 3:** Logistic regression analysis of different degrees of body fat distribution characteristics and hyperuricemia in MAFLD

variable	OR (95% CI) P			
	**Model1**	**Model2**	**Model3**	**Model4**
**Total body fat mass**	1.000 (1.000, 1.000) = 0.896	1.000 (1.000, 1.001) = 0.241	1.000 (1.000, 1.000) = 0.918	1.000 (1.000, 1.000) = 0.671
**Upper limbs fat mass**	1.000 (0.999, 1.001) = 0.953	1.000 (0.999, 1.002) = 0.654	1.000 (0.999, 1.001) = 0.729	1.000 (0.999, 1.001) = 0.532
**Trunk fat mass**	1.000 (1.000, 1.000) = 0.948	1.000 (1.000, 1.001) = 0.400	1.000 (1.000, 1.000) = 0.899	1.000 (1.000, 1.000) = 0.961
**VAT**	1.016 (1.004, 1.027) = 0.006	1.018 (1.004, 1.032) = 0.009	1.015 (1.002, 1.027) = 0.023	1.014 (1.001, 1.028) = 0.032
**SAT**	1.003 (0.996, 1.010) = 0.367	1.006 (0.997, 1.014) = 0.177	0.999 (0.992, 1.006) = 0.856	1.000 (0.993, 1.007) = 0.991
**Android fat mass**	1.000 (0.998, 1.002) = 0.798	1.001 (0.998, 1.003) = 0.660	1.000 (0.999, 1.001) = 0.643	0.999 (0.998, 1.001) = 0.402
**Android/Gynoid ratio**	0.063 (0.000, 37.604) = 0.396	0.117 (0.000, 342.858) = 0.599	0.123 (0.001, 15.854) = 0.398	0.003 (0.000, 1.506) = 0.067
**Adipo-IR**	1.089 (1.017, 1.166) = 0.014	1.079 (1.006, 1.157) = 0.033	1.100 (1.010, 1.198) = 0.028	1.094 (1.002, 1.194) = 0.046

Model1: adjusted for sex, age, hypertension, BMI; Model2: adjusted sex, age, BMI, HbA1c; Model3; adjusted sex, age, BMI, HOMAIR; Model4:adjusted sex, age, BMI, TG, TC, HOMAIR

*Abbreviations*: *VAT* visceral adipose tissue, *SAT* subcutaneous adipose tissue, *Adipo-IR* adipose tissue insulin resistance

### Prevalence of MAFLD with or without HUA across the quartiles of VAT and SAT

Furthermore, to explore whether the prevalence rates of MAFLD with or without HUA increased with increasing abdominal fat, we further divided VAT and SAT into quartiles. As predicted, the percentage of MAFLD with HUA increased significantly with increasing quartiles of VAT or SAT, while the percentage of normal people significantly declined with increasing abdominal fat, as determined by the Cochran–Armitage trend test (all *p* < 0.05). However, no such association was observed in the case of participants with MAFLD with normal SUA (Table 4).

**Table 4:** Proportion of patients in different groups across quartiles of VAT and SAT

Items	Normal population	MAFLD with normal SUA	MAFLD with high SUA	*P* values
V1n (%)	43.00 (58.90)	42.00 (17.60)	8.00 (7.30)	
V2	19.00 (26.00)	68.00 (28.60)	16.00 (14.70)	
V3	9.00 (12.30)	71.00 (29.80)	33.00 (30.30)	
V4	2.00 (2.70)	57.00 (23.90)	52.00 (47.70)	< 0.000
S1	42.00 (58.30)	50.00 (21.00)	7.00 (6.40)	
S2	15.00 (20.80)	71.00 (29.80)	22.00 (20.20)	
S3	10.00 (13.90)	68.00 (28.60)	30.00 (27.50)	
S4	5.00 (6.90)	49.00 (20.60)	50.00 (45.90)	< 0.000

*Abbreviations*: *VAT* visceral adipose tissue, *SAT* subcutaneous adipose tissue, *V1* the first quartile of VAT, *V2* the second quartile of VAT, *V3* the third quartile of VAT, *V4* the fourth quartile of VAT, *S1* the first quartile of SAT, *S2* the second quartile of SAT, *S3* the third quartile of SAT, *S4* the fourth quartile of SAT

### Changes in SUA after decreasing SAT and VAT in MAFLD

We also assessed the changes in SUA in 44 weight-loss MAFLD patients. As shown in Table 5, after weight loss, SUA significantly reduced (before 396.41 ± 118.80 vs after 362.52 ± 104.72; *p* < 0.05). Furthermore, the changes in SUA levels were significantly correlated with changes in body fat (Table 6), especially VAT (*r* = 0.32, *p* < 0.05), SAT (*r* = 0.40, *p* < 0.01), truck fat (*r* = 0.97, *P* < 0.01), and android fat (*r* = 0.89, *p* < 0.01), but not thighs fat mass (*p* > 0.05), gynoid fat (*p* > 0.05), and upper limbs fat mass (*p* > 0.05) (Table 6).

**Table 5:** Main clinical characteristics of study patients at baseline and post lost weight

Characteristics	Before lost weight (*n* = 44)	After lost weight (*n* = 44)	Change (Δ)	*P* values
BMI (kg/m^2^)	28.53 ± 4.21	26.90 ± 4.11	-1.63 ± 2.22	< 0.000
Weight (Kg)	76.80 ± 14.65	72.60 ± 15.64	-4.20 ± 5.46	< 0.000
SUA (umol/L)	396.41 ± 118.80	362.52 ± 104.72	-33.89 ± 95.88	0.024
ALT (U/L)	30.00 (21.00,52.00)	20.50 (17.00,32.75)	-17.03 ± 36.86	0.014
AST (U/L)	22.00 (16.00,38.00)	19.00 (15.00,26.00)	-8.06 ± 19.98	0.038
ALP (U/L)	74.00 (64.50,84.00)	59.00 (53.25,70.50)	-13.11 ± 26.59	0.001
VAT (cm^2^)	95.64 ± 38.70	74.18 ± 32.77	-21.45 ± 31.25	< 0.000
SAT (cm^2^)	235.75 ± 82.82	199.26 ± 81.72	-36.49 ± 44.61	< 0.000
Total body fat (%)	41.31 ± 3.20	37.14 ± 5.29	-4.18 ± 3.70	0.015
Upper limbs fat mass (g)	3318.86 ± 748.28	3159.14 ± 935.10	-159.71 ± 636.79	0.532
Thighs fat mass (g)	8195.86 ± 2300.37	6635.29 ± 2274.57	-1560.57 ± 844.03	0.003
Trunk fat mass (g)	16,334.14 ± 3285.92	12,711.14 ± 3868.50	-3623.00 ± 1834.48	0.002
Total body fat (g)	29,032.57 ± 5490.54	23,178.57 ± 6554.79	-5854.00 ± 2828.46	0.002
Android fat mass (g)	2597.43 ± 569.47	1880.00 ± 650.49	-717.43 ± 297.57	0.001
Gynoid fat mass (g)	3589.14 ± 1056.64	3589.14 ± 1056.64	-871.43 ± 364.11	0.001

*Abbreviations*: *BMI* body mass index, *SUA* serum uric acid, *ALT* alanine aminotransferase, *AST* aspartate aminotransferase, *ALP* alkaline phosphatase, *VAT* visceral adipose tissue, *SAT* subcutaneous adipose tissue

**Table 6:** Correlation of ΔSUA and changes of body fat distribution

Items	r	*P* Values
ΔVAT	0.32	**0.036**
ΔSAT	0.40	**0.006**
ΔTrunk fat mass	0.97	** < 0.000**
ΔThighs fat mass	0.45	0.317
ΔUpper limbs fat mass	-0.35	0.445
ΔTotal body fat	0.96	**0.001**
ΔAndroid fat mass	0.89	**0.008**
ΔGynoid fat mass	0.74	0.055

*Abbreviations*: *VAT* visceral adipose tissue, *SAT* subcutaneous adipose tissue

## Discussion

In the present study, we uncovered the correlation between SUA and fat distribution in MAFLD patients. Our findings may supplement previous research and have therapeutic implications in both HUA and MAFLD in clinical practice. We established that abdominal fat promotes the co-existence of HUA and MAFLD, VAT and adipo-IR are independent risk factors for HUA in MAFLD, and weight loss, especially, decreasing VAT, is important in lowering SUA and managing MAFLD. To the best of our knowledge, this is the first study to investigate the association between body fat distribution and SUA in patients with MAFLD.

The present study showed there were no significant differences in NFS, FIB-4, and CAP between MAFLD patients with normal SUA or with HUA, which implies that fat deposition status and fibrosis indices were comparable between the two MAFLD groups. However, MAFLD patients with HUA had much higher fasting insulin, OGIRT AUC, VAT, SAT, Adipo-IR, trunk fat mass, and android fat mass than those with normal SUA, indicating that MAFLD patients with HUA are characterized by more central fat and insulin resistance. Furthermore, with increasing quartiles of SUA, fat accumulation in central deposits like the abdomen, trunk, and android increased; this is also a feature of insulin resistance, especially, adipose tissue insulin resistance. Previous studies showed that elevated serum uric facilitates insulin resistance-mediated accumulation of visceral fat [38, 39]. Whereas low SUA levels with anti-hyperuricemia promote overall metabolic conditions and loss of VAT [20, 40]. The role of increased SUA appears to be greater when considering Adipo-IR as a promoter of VAT accumulation [38]. Increased intracellular SUA levels upregulate lipogenesis-related proteins directly and dose-dependently and downregulate the expression of lipolysis-related proteins and thus, induce excessive TG accumulation in adipocytes [41]. UA promotes citrate conversion to acetyl-CoA for de novo lipogenesis in hepatocytes [42], leading to an increase in circulating FFA. Elevated FFA in adipocytes further converts to TG and finally deteriorates visceral obesity. These mechanisms explain why HUA correlated strongly with VAT in MALFD patients.

In the present study, we found that both HOMA-IR and Adipo-IR were significantly higher in patients with MAFLD than the controls; whereas only Adipo-IR was higher in MAFLD patients with HUA than those with normal SUA. Most importantly, Adipo-IR increased with increasing SUA. While HOMA-IR is a general index of IR, the Adipo-IR index has been widely used as a simple, unique, and reliable predictor of IR in adipose tissue in metabolic disorders associated with obesity [43–47]. Adipo-IR is reported to estimate insulin resistance in adipose tissue reasonably well compared with a gold standard clamp with FFA tracers [43]. Although the two indices are highly correlated with each other theoretically and practically [34, 48], a discordance between them for indicating metabolic diseases has been reported [49, 50]. Adipo-IR was reported to link with hypertriglyceridemia and visceral adiposity more closely [50], which is in accordance with our result. Furthermore, Adipo-IR is more related to the severity of liver fibrosis compared to HOMA-IR [51]. We also found that Adipo-IR is an independent risk factor for HUA in MAFLD patients adjusted for HOMA-IR, BMI, TG, and TC. Moreover, weight loss improved Adipo-IR and HUA in MAFLD patients. Therefore, the role of adipose tissues in MAFLD with HUA is more pathogenic, and more attention should be paid to Adipo-IR when considering HUA in MAFLD patients.

Notably, in this study, SUA levels in MAFLD patients were back to normal after weight loss, without the use of anti-HUA drugs. It is well established that weight gain has been strongly correlated with increasing SUA levels [52, 53] and weight loss can reduce the levels of SUA [54, 55]. Renal excretion of urate could be reduced in condition of Obesity [56, 57]. Therefore, increased SUA levels are reportedly an outcome and not a cause of obesity [58], and obesity is assumed to lead to high SUA levels and thus, cause decreased inflammatory responses, endothelial dysfunction, nitric oxide production, and enhanced oxidative stress [59]. Previous studies have shown that obese individuals have a significantly higher risk of gout than those with normal weight, regardless of sex and race [60–63]. Furthermore, a recent study estimating the correlation between weight change and obesity and the incidence of gout in a retrospective US adults study concluded that participants with a stable obese BMI gaining weight who were non-obese previously were consistently associated with increased risk of gout. This strengthened the importance of keeping non-obese weight during adulthood, especially those obese individuals who got weight loss, to reduce gout risk continuously in their later life [64]. Therefore, we postulate that the main consideration for controlling HUA in obese individuals must be weight loss and not anti-HUA drugs. It is significant for obese HUA patients to maintain weight within the normal range over using anti-HUA drugs alone. Therefore, a normal body fat percentage is important for both HUA and future gout.

The strengths of our study are as follows. First, we assessed fat distribution in the light of SUA in MAFLD patients for the first time and showed that VAT and Adipo-IR were the independent risk factors of SUA in MALFD. Second, we tested the hypothesis that weight loss is more important in decreasing SUA in MAFLD than anti-HUA drugs and that keeping body weight within the normal range may reduce the risk of recurrence of HUA and future gout associated with increased weight.

There are several limitations to our study. First, we use ultrasonography and fibro scan to diagnose MAFLD. Although [1H]-MRS is noninvasive and considered the most accurate method, ultrasonography is sufficient correlated with MRS. Furthermore, although ultrasonography may underestimate fat contact, it does show better specificity. Second, the sample size is relatively small and limited our ability to account for potential confounders during the analysis. However, the present study is sufficient to show novel associations between body fat distribution and SUA in MAFLD patients. Additionally, this case–control study cannot prove causality, however, we carried out a pilot study to show that the extent of changes in body fat was correlated with a decrease in SUA in MAFLD. This phenomenon should be validated in future clinical studies.

## Conclusions

This study is valuable in that it is the first study to assess the relationship between changes in SUA levels and changes in visceral fat in MAFLD patients. Supplementary to previous studies, the present study also shows that weight loss is of great importance in lowing SUA, as opposed to anti-HUA drugs in MAFLD patients. More importantly, VAT and Adipo-IR instead of other fat compartments were established as independent risk factors of HUA in MAFLD patients. We suggest that more attention should be paid to the role of excessive VAT in the development and occurrence of HUA in MAFLD patients.

## Data Availability

Data will be made available on reasonable request to the corresponding author.

## Abbreviations

NAFLD: Nonalcoholic fatty liver disease
BMI: Body mass index
WHR: Waist hip ratio
SBP: Systolic blood pressure
DBP: Diastolic blood pressure
Ogirt AUC: Area under Ogirt curve
HbA1c: Glycosylated hemoglobin A1c
ALT: Alanine aminotransferase
AST: Aspartate aminotransferase
TC: Total cholesterol
TG: Triglyceride
LDL-c: Low-density lipoprotein cholesterol
HDL-c: High-density lipoprotein cholesterol
SUA: Serum uric acid
VAT: Visceral adipose tissue
SAT: Subcutaneous adipose tissue
NFS: Non-alcoholic fatty liver disease fibrosis score
FIB-4: Fibrosis-4 score
HOMA-IR: Homeostasis model assessment of insulin resistance
CAP: Controlled attenuation parameters of liver
FFA: Free fatty acid
Adipo-IR: Adipose tissue insulin resistance
FPG: Fasting plasma glucose
MAFLD: Metabolic dysfunction associated with fatty liver disease
